# Propolis-based niosomes as oromuco-adhesive films: A randomized clinical trial of a therapeutic drug delivery platform for the treatment of oral recurrent aphthous ulcers

**DOI:** 10.1038/s41598-018-37157-7

**Published:** 2018-12-21

**Authors:** Mona G. Arafa, Dalia Ghalwash, Dina M. El-Kersh, M. M. Elmazar

**Affiliations:** 1 Department of Pharmaceutics and Pharmaceutical Technology, Faculty of Pharmacy, The British University in Egypt (BUE), El-Sherouk city, Cairo 11837 Egypt; 2 Department of Oral Medicine and Periodontology, Faculty of dentistry, The British University in Egypt (BUE), El-Sherouk city, Cairo 11837 Egypt; 3 Department of Pharmacognosy, The British University in Egypt (BUE), El-Sherouk city, Cairo 11837 Egypt; 4 Department of Pharmacology, Faculty of Pharmacy, The British University in Egypt (BUE), El-Sherouk city, Cairo 11837 Egypt; 5 Chemotheraputic Unit, Mansoura University Hospitals, Mansoura, 35516 Egypt

## Abstract

Oromuco-adhesive films for buccal delivery of Propolis extract (PPE) entrapped in niosomes, were prepared to treat oral recurrent aphthous ulcer (RAU). PPE was investigated for antimicrobial compounds. Niosomes composed of span60 and cholesterol were evaluated for particles size, polydispersity index (PDI), zeta-potential, entrapment efficiency and *in vitro* release. The formed oromuco-adhesive films containing niosomal PPE were evaluated for swelling, mucoadhesion and elasticity. 24 patients suffering from RAU were divided equally into medicated and placebo groups and participated in this study to examine the onset of ulcer size reduction, complete healing and pain relief. Ultra-performance liquid chromatography-high resolution mass spectrometry revealed the presence of pinocembrin, pinobanksin, chrysin and galangin as antimicrobial flavonoids with total content of 158.7 ± 0.15 µg quercetin equivalents and phenolic content of 180.8 ± 0.11 µg gallic acid equivalents/mg. Multilamellar niosomes of 176–333 nm displayed entrapment efficiency of 91 ± 0.48%, PDI of 0.676 and zeta potential of −4.99. *In vitro* release after 8 h from niosomal dispersion and films were 64.05% and 29.09 ± 0.13% respectively. Clinical results revealed duration of film adherence from 2–4 h in the two groups. The onset of ulcer size reduction in medicated group was attained within second and third day, complete healing was achieved within first 10 days of treatment and pain relief lasted for more than 4–5 h, in contrast to the placebo group. This oromuco-adhesive films which offer controlled and targeting drug delivery can be proposed as a new therapeutic strategy in the treatment of oral recurrent aphthous ulcer.

## Introduction

Transmucosal drug delivery is achieved *via* the mucosa of the oral cavity, which is considered a readily accessible route of administration^1^. Therefore, the concept of muco-adhesion in the oral cavity has received considerable interest in formulation science^2^. In addition to advantages related to rapid systemic effects, such as avoiding stomach acidity and the first-pass effect^3^, oromuco-adhesive films also have local effects that are achieved by localizing drugs to the target site^4^ and thus improving and prolonging drug contact with the mucosa. In addition, such drugs can be administered easily, with ease of access and good patient compliance, making them suitable substitutes for nauseous patients, in addition to paediatric and geriatric patients, thereby improving and enhancing the bioavailability of drugs. Many muco-adhesive films have been formulated to treat oral infections^5–7^, such as recurrent aphthous ulcers (RAUs), which are considered the most common and most painful oral mucosal lesions, with a wide range of reported prevalence (from 5 to 25%) in different populations^8^. RAUs are most frequently encountered in patients between 10 and 40 years of age; they primarily affect females, and they are more prevalent in people with higher socioeconomic levels^9^. Three clinical subtypes are well recognized: major, minor and herpetiform RAUs. The most commonly encountered presentation of the disease is the minor type, representing 70–85% of all cases^8^. This type of RAU is characterized by the appearance of initially necrotic, recurrent, painful and rounded or oval ulcers with well-defined borders surrounded by an erythematous halo. It causes severe pain, in addition to decreasing quality of life, as the patient cannot swallow, drink, eat or even speak^8^.

RAUs are usually of unknown aetiology, but several predisposing and risk factors have been implicated in the pathogenesis of RAUs, such as genetic predisposing factors, trauma, stress, haematologic disorders, microbial or immunologic factors and hormonal disturbance^10^. Since no specific aetiology has been identified, the treatment of RAUs is non-specific and symptom based.

Topical therapeutic agents have been used in the management of RAUs, including various anti-inflammatory agents, immunomodulatory agents, antimicrobials and analgesic drugs^11–13^. Some topical corticosteroid preparations, such as triamcinolone in orabase, have been used in the management of RAUs. Chlorhexidine- or benzydamine-based mouth rinses are also used to reduce the duration of ulcers and relieve the symptoms of RAUs^14,15^.

Several approaches have been used to obtain oromuco-adhesive dosage forms as a platform for controlled delivery of drugs^16^. Nanovesicles have attracted great attention in the context of topical applications because they have tremendous benefits, such as prolonging the therapeutic effect of the drug by acting as drug reservoirs. They can also entrap both hydrophilic and hydrophobic drugs and act as local depots for the sustained release of oromucosal active agents, in addition to their ability to enhance permeation^17^. Nanovesicles, such as niosomes, that are prepared from nonionic surfactants with cholesterol are characterized by high stability with a strong potential to improve drug bioavailability; they act as skin penetration enhancers^18^. Niosomes are considered appropriate carriers for anti-infective and anticancer drugs^19^. They also augment targeted drug delivery, improve oral bioavailability of poorly absorbed drugs, minimize drug toxicity and improve drug therapeutic indices^20,21^. These findings are consistent with many reported studies of niosomal formulations of different therapeutic agents that showed a gradual increase in drug activity due to controlled release, especially when the entrapped drug is hydrophilic^22–25^. The inclusion of nanovesicles as niosomes in muco-adhesive films increases drug uptake, decreases skin irritation and avoids the first-pass effect, which may be attributed to the deep penetration of nanoparticles into the human body due to their particle size and surface properties^26–28^.

Natural products are a promising source for the innovation of new therapeutic agents, as they have fewer adversative properties. In recent decades, the bee product propolis has attracted interest from researchers, as several studies have been conducted to investigate the biological properties of propolis and explore its potential for the development of new drugs^29–31^.

Propolis is a sticky substance composed of resin, wax and essential oils that is prepared by bees (*Apis mellifera*) from the exudates of trees and flowers to fill up the holes in their hives. It has been well known since ancient times in folk medicine as an important bioactive food supplement and an excellent preservative with antibacterial and antifungal activity^32^. The main pharmacological activities of propolis are its anti-inflammatory, antimicrobial, antioxidant, immuno-stimulant and wound healing activities^33,34^. Although the active constituents of propolis differ according to the geographical source, there are still common major classes of propolis preparations, such as phenolic acids and flavonoids, which are considered the primary biologically active constituents^32^. Previous phytochemical analyses that detected these main constituents for the aforementioned uses reported the presence of the phenolic acids *viz*. cinnamic acid, caffeic acid, *p*-coumaric acid and ferulic acid as well as flavonoids *Ca*. quercetin, pinobanksin, genistein, luteolin, kaempferol, chrysin, galangin and pinocembrin^33,35^.

In the present work, the inclusion of propolis extract (PPE) in niosomal oromuco-adhesive films is of great importance because the current dosage forms of propolis, such as suspensions^36^, buccal pastes^37^, tinctures^38^, solid powders^39^, and ethanolic extracts^40^, have not been enhanced to deliver PPE in a controlled manner with good mucosal permeation profiles to augment bioavailability.

The new therapeutic strategy proposed here is to treat aphthous ulceration by maintaining a satisfactory therapeutic level of the active ingredient in the mouth for a prolonged duration of time and improving drug absorption, accompanied by increased patient acceptance and compliance.

## Methods and Materials

### Materials

Commercial Propolis was supplied from Imtenan Health Co., Egypt. Cholesterol (95% stabilized) was purchased from Acros organics, U.K., Span 60, chloroform and polyvinyl alcohol (PVA, CAS: 9002-89-5, MW:89, 000–98,000, 99% hydrolyzed) were purchased from Sigma-Aldrich, Germany. Hydroxy Propyl methylcellulose (HPMC, USP grade, CAS: 9004-65-3, 2600–5600 cP) and Eudragit L-100 were kindly supplied from Cairo Pharmaceuticals Co., Egypt. Monopotassium dihydrogen phosphate and sodium phosphate dibasic anhydrous were bought from Adwic, Al Nasr Pharmaceutical Chemicals.co. Egypt. Cellophane membrane; Spectra/por dialysis membrane 12000–14000 Mwt cut off was used. Authentic quercetin, gallic acid and Folin-Ciocalteu reagent were obtained from Sigma Aldrich (Germany). Acetonitrile and formic acid were obtained from Fluka Analytical, LC-MS Chromasolv.

### Instruments

PH-meter (CA 92634, Beckman Instruments Fullerton, USA), Transmission electron microscope (JEOL JSM-6510 LV., Tokyo, Japan), Scanning electron microscope (JEOL 5500 LV., Tokyo, Japan), Rotary evaporator (OSB-2100, N-1200A, Shanghai Eyela Co. Ltd., China), Freeze centrifuge (2-16KL, Sigma Laborzentrifugen GmbH, Germany) and Malvern Instruments Ltd (Zetasizer Nano-Zs90, MPT-Z, UK) were used. UV visible spectrophotometer Schimadzu (U.V-1650PC) was used for the content of flavonoid and phenolic compounds. Calibrated Orbitrap Elite mass spectrometer (Thermo Scientific, Germany) by the Pierce ESI negative ion calibration solution (product No. 88324, Thermo Scientific, Germany) and equipped with a heated electrospray ion source of −3 KV spray voltage and coupled to a Thermo Scientific ultra performance liquid chromatography- photodiode array detector-high resolution mass spectrometry (UHPLC-PDA-HRMS) system (Dionex UltiMate 3000, Germany). Reversed phase column (C-18, Waters), 1.8 µm particle size column (150 × 1 mm i.d, pore size 100 Å). Thermo Fisher Scientific photodiode array (PDA) detector (220–600 nm).

### Preparation of PPE

Three grams of dried propolis powder was extracted using ethanol: water (80:20). The hydroalcoholic PPE was concentrated on a rotary evaporator under reduced pressure and used for identification of the antimicrobial flavonoids using ultra performance liquid chromatography- photodiode array detector-high resolution mass spectrometry (UPLC-PDA-HRMS), determination of the total flavonoid and phenolic contents^41^.

### Determination of the content of total flavonoid and phenolic compounds in PPE

The content of total flavonoid compounds was determined by an AlCl_3_ colorimetric assay^42^ using quercetin as a standard. The content of total phenolic compounds was determined by the Folin-Cio-Calteau assay^43^ using authentic gallic acid to prepare the calibration curve.

### Preparation of niosomal PPE

Niosomes containing phenolic compounds from PPE (F1, Table 1) were prepared by the reversed phase evaporation method (REV). A mixture of Span 60 and cholesterol (1:1 molar ratio) was dissolved in chloroform. Subsequently, PPE (ethanol:water, 80:20) equivalent to 0.5 g of phenolic compounds was added and mixed for 1 min. The mixture was then emulsified using a bath sonicator for 10 min at 10 °C. The emulsion was rotary-evaporated at 40 °C with a rotation speed of 50 rpm for approximately 15 min to remove the organic solvent. Traces of chloroform and ethanol were eliminated by employing a rotary evaporator for an additional 10 min. The suspension was centrifuged using a refrigerated centrifuge at 4 °C and 10,000 rpm for 1 h, lyophilized and stored at 4 °C for further investigations.

**Table 1:** Composition of niosomal PPE and oromucoadhesive film containing niosomal PPE.

Formula	Ingredients					
	PPE(%)	Span 60(%)	Cholesterol (%)	Eudragit L-100 (%)	HPMC (5%) (%)	PVA (4%) (%)
F1	10	53	47	—	—	—
F2	1.5	—	—	25	50	25

### Characterization of niosomal PPE

#### Scanning electron microscopy (SEM)

Samples were scattered on an SEM sample holder with double-sided sticky tape, coated with 150 Å gold for two minutes by an SPI Module^TM^ gold sputter coater and examined using the high-vacuum mode of SEM.

#### Transmission electron microscopy (TEM)

A 10-fold diluted aqueous droplet of the niosomal dispersion was subjected to a collodion-coated 300 sieve copper grid, left for 5 min, and adsorbed using filter paper; a drop of aqueous uranyl acetate (2%) was then applied. The remaining solution was eliminated, and the samples were left in air to dry and then tested at 80 kV^44^.

#### Particle size, distribution and zeta potential of niosomal PPE

The size, distribution and zeta potential of the formed niosomes were determined using a Zeta Potential Analyzer (Malvern Zetasize Nano-zs90, Malvern Instruments, Malvern, UK). The scattering angle was adjusted to 90 degrees, the size distribution was investigated at room temperature, and the samples were appropriately diluted with dispersant water. The mean diameter ± standard deviation of 3 determinations was calculated.

#### Entrapment efficiency (EE%) of PPE in niosomes

Two milligrams of niosomal PPE was suspended in absolute alcohol, and 0.1 ml of the suspension was then diluted with another 5 ml of absolute alcohol and placed in a bath sonicator for ten minutes to obtain a clear solution. The concentration of entrapped PPE was determined spectrophotometrically at 277 nm using a UV spectrophotometer and then compared to a sample prepared from empty niosomes that were treated using the same procedures. The amount of drug entrapped inside the niosomes was obtained from the following equation:

1 $${\rm{EE}} \% ={\rm{ED}}\ast 100{\boldsymbol{/}}{\rm{TD}},$$

where: EE% is the percent of entrapment efficiency, ED is the concentration of drug that was entrapped inside niosomes and TD is the theoretical concentration of drug.

### *In vitro* release of niosomal PPE

The release of PPE from niosomes was determined using the membrane diffusion technique. One millilitre of phosphate buffer (pH 6.8) was used to suspend niosomal PPE equivalent to 5 mg of phenolic compounds. The suspension was transferred to a glass tube that had a soaked cellulose membrane with a closed lower end. The glass tube was placed in a beaker containing 50 ml of phosphate buffer (pH 6.8) that was maintained at 37 °C. Using a shaker water bath, the beaker was kept shaking at 50 rpm. Aliquots were collected at time intervals for 8 h, and the drug concentration was then determined spectrophotometrically at 277 nm. The experiment was performed in triplicate. The obtained data were kinetically analysed to determine the pattern of drug release^45^.

### Formulation of niosomal PPE oromuco-adhesive films

Oromuco-adhesive films containing niosomal PPE (F2, Table 1) were prepared by a solvent casting technique^46^ using a polymer combination of Eudragit L-100 (EU-L100), hydroxypropyl methylcellulose (HPMC) and polyvinyl alcohol (PVA). Briefly, Eudragit L-100 (95%) was dissolved in ethanol and mixed with HPMC (5%) and PVA (4%). To this mixture, 2 ml of propylene glycol (PG) was added, and the mixture was stirred for 1 h using a magnetic stirrer until a clear, homogeneous, bubble-free solution was obtained. To this mixture, niosomal PPE corresponding to 0.45 g of PPE was added, and the solution was mixed thoroughly. The formed mixture was then transferred into Teflon-coated round dishes. The formed films were dried at 25 °C for 120 min and then dried for an additional 18 h at 40 °C in a hot air oven. Finally, the films were dried under vacuum using a vacuum desiccator for 4 h at room temperature. The dried films were carefully removed and stored for further examination.

### Characterization of the niosomal PPE oromuco-adhesive films

#### Determination of bioadhesive force

A piece of chicken pouch mucosa was placed on top of each glass bottle and fixed with an elastic screw. Next, one of the previously prepared bottles (bottle 1) was attached to the balance, and a second bottle was located on an adjustable pan (bottle 2). A 2 cm^2^ oromuco-adhesive film was adhered onto bottle 1, and the height of bottle 2 was adjusted so that the film was placed between the mucosal membranes of both bottles^47^.

Additional weights were added until the two bottles were attached. Water kept in a burette was dripped at a constant rate of 10 mg/sec into a beaker placed under the burette. The two bottles eventually detached as a result of the gradual increase in the weight of the water. The force used to disconnect the two bottles (dyne/cm^2^) was calculated from the following equation^48^:

2 $${\rm{F}}=({\rm{Ww}}\times {\rm{g}})\,/\,{\rm{A}},$$

where: F = The biodhesive force (dyne/cm^2^).

Ww = The weight of water (gm).

g = The acceleration due to gravity (cm/sec.^2^).

A = The surface area of mucous membrane (cm^2^).

The experiment was carried out in triplicate for each formulation and the mean bioadhesive force was determined.

#### Swelling study

The weight and diameter of the formed film were determined, and the samples were left on the top of an agar dish to swell, while the whole setup was placed in an incubator that was kept at 37 ± 0.5 °C. The increase in the mass and diameter of the films (*n* = 3) was calculated at 60:300 min. Percent swelling (% *S*) was determined by the following equation:

3 $$ \% {\rm{S}}={\rm{Xt}}-{\rm{X0}}\,\ast \,{\rm{100}}\,/\,X0$$

where: %S = percent swelling,

Xt = mass or diameter of the puffy film after time t,

X0 = the initial film mass or diameter at t_0_.

#### Surface pH

Oromuco-adhesive films were allowed to increase in volume for 2 h on the uppermost layer of an agar dish. A pH meter was located on top of the swollen film and used to measure the pH of its surface. This process was repeated in triplicate.

#### Film thickness

The thickness of the 2 cm^2^ circular oromuco-adhesive film was measured using a standard screw meter at three different points on the film. The experiment was carried out in triplicate, and the median was computed.

#### Weight uniformity

A randomly selected 2 cm^2^ circular film was accurately weighed using an electronic balance, and the weight uniformity was then evaluated.

#### Folding endurance

The folding endurance of the 2 cm^2^ circular films was tested using a single film that was repeatedly folded at the same point until it was disrupted or bent 300 times without breaking. The mean values of three determinations were recorded.

#### Drug content

Drug content was calculated as follows: three 2 cm^2^ films were solubilized in 10 ml of phosphate-buffered saline (PBS) (pH 6.8) using a digital shaking water bath for 120–180 min at 37 ± 0.5 °C, with shaking at 50 rpm. The resulting solution was suitably diluted. The concentration of PPE was determined spectrophotometrically at 277 nm^6,49^.

### *In vitro* release of niosomal PPE oromuco-adhesive films

A standard Elmasonic paddle apparatus (S60 H, Elma Hans Schmidbauer GmbH, Germany) was used to determine the drug release profile. A 2 cm^2^ film (equivalent to 5 mg of drug) was attached with sticky tape and fixed to the central shaft. After 2 min, the bowl of the apparatus was filled with PBS (pH 6.8 to simulate salivary fluid) and kept at 37 ± 0.5 °C with stirring at 50 rpm. For each film sample, at predetermined time points of 1, 2, 3, 4, 5, 6, 7 and 8 h, aliquots were collected and replaced by the same volume of freshly prepared medium maintained at the same temperature. The released amounts of PPE were examined spectrophotometrically at 277 nm^50^.

### *Ex vivo* permeation of the niosomal PPE oromuco-adhesive films

The oromucosal permeation of PPE was measured across excised chicken pouch mucosa using Franz diffusion cells. All animal work was conducted in accordance with the guidelines outlined in the *Guide for the Care and Use of Laboratory Animals* and was approved by the Ethical Committee of the British University in Egypt. The mucosa was cleaned and washed with simulated saliva (pH 6.8 ± 0.05). The pouch mucosa was fixed between the receptor and donor chambers. The film (2 cm^2^, equivalent to 5 mg of drug) was pressed on the mucosal side, and the compartments were clamped together. One millilitre of simulated saliva was added to the donor chamber as a wetting agent. Phosphate buffer at pH 6.8 was added to fill the receptor compartment. The diffusion cell was kept at 37 ± 0.5 °C and stirred at 100 rpm, and these parameters were also applied to the receptor compartment^51^. At predetermined time points of 1, 2, 3, 4, 5, 6, 7 and 8 h, aliquots were collected and replaced by freshly prepared PBS. The permeated amounts of PPE were examined at 277 nm. The permeation flux was computed using the following equation^52^:

4 $${\rm{Jss}}\,=\,{\rm{dQ}}\,/\,{\rm{dt}}\,\ast \,{\rm{A}},$$

where: Jss = steady-state permeation flux (μg/cm^2^/h), A (cm^2^) = the area of skin tissue where drug permeate through t. (dQ /dt) = the amount of drug passing *via* skin / unit time at a steady state.

The propolis permeability coefficient (P) across chicken pouch mucosa was calculated using the relation derived from Fick’s first law of diffusion as follows: P = Jss/Cd where, P is the permeability coefficient (cm s^−1^) and Cd is the donor drug concentration (µg ml^−1^).

#### Kinetic release study

*In vitro* release data were analysed using linear regression according to zero-order, first-order and Higuchi diffusion models. Additionally, the Korsmeyer–Peppas model was determined using the following equation^53^:

5 $${\rm{Mt}}/\,M\infty \,=\,\mathrm{ktn},$$

where: Mt = the amount of drug released at time t, M∞ = the amount of drug released as time approaches infinity, k = a constant incorporating characteristics of the particle system or network, n = the diffusion exponent.

### Clinical study of the niosomal PPE oromuco-adhesive films

The current clinical study was congruent with the ethical principles conveyed in the 2002 version of the Helsinki Declaration and accepted by the Ethical Committee of the Faculty of Pharmacy at The British University in Egypt. The present study had a parallel design with an allocation ratio of 1:1 and was performed on a total of 24 patients suffering from RAUs. The patients were randomized into two groups using a computer-generated sequence with random allocation software. The two groups received oromuco-adhesive film of the same shape and colour, but the first group was treated with the niosomal PPE oromuco-adhesive film (medicated group), and the second group received the film without the active component (placebo group). Placebo and intervention participants were recruited from the outpatient Clinic of the Faculty of Dentistry at The British University in Egypt during a period of 3 months. Patient informed consent was obtained after discussing the protocol details with each patient and after obtaining his/her agreement to sign the consent form.

The patients were subjected to a thorough clinical examination and history by an oral medicine specialist to diagnose the oral ulcers; all patients were thoroughly informed about the study. Only patients with oral ulcers within the first day to the third day after appearance were included in the present study.

The exclusion criteria in the present study were the presence of systemic or local disease, such as hepatitis, cardiac conditions, hypertension, diabetes mellitus, renal problems, mental disorders and AIDS. In addition, patients were excluded if they had used any immunosuppressive drugs or systemic corticosteroids during the past three months or any local steroid treatment in the past month, if they were using any nutritional supplements, folic acid, or antioxidants, or if they were pregnant or nursing.

All patients were asked to avoid using antiseptics, antibiotics or analgesics during the study, as such products might aid in curing the oral ulcers. The patients were blinded to whether they received propolis or placebo, and they were warned about the potential for allergic responses in some patients; in such cases, the patients were instructed to immediately terminate use of the film and report the reaction.

The patients were first instructed on how to apply the 2 cm^2^ muco-adhesive film to the oral ulcer by applying light force with a fingertip for 20 s. The patients were asked to apply the film two times per day and to refrain from eating for at least one hour after film application on the oral ulcer.

The patients were handed a checklist for monitoring improvements in clinical parameters, which included the duration of disappearance of pain after film application (hours), the time to complete healing of the aphthous ulcer (weeks), the onset of ulcer size reduction after film application (days), the duration of propolis film adherence onto the oral mucosa (hours) and the level of patient satisfaction on a scale from 1–10. After total healing of the oral RAU, the checklists were collected from patients, the results were tabulated, and statistical analysis was conducted.

### Statistical analysis

The results of *in vitro* and *ex vivo* experiments were analysed using ordinary non-linear regression and a paired Student’s t test. Clinical experiments were compared using the Chi square test. A p-value of <0.05 was considered statistically significant. Statistical analysis was performed using Prism-5 (Graph Pad Software Inc., Demo. Ink).

### Ethics Approval

All clinical work was conducted in accordance with the ethical regulations of World Medical Association Declaration of Helsinki (1996) after the approval of the ethical committee of Faculty of Pharmacy, The British University in Egypt (BUE).

## Results

### The content of total flavonoid and phenolic compounds

The total flavonoid and phenolic contents were 158.7 ± 0.15 µg quercetin equivalents (QE) and 180.8 ± 0.11 µg gallic acid equivalents (GAE) per mg of PPE sample, respectively.

### Identification of antibacterial flavonoids using UPLC-PDA-HRMS

In the current study, the detection of major flavonoids, which could be attributed to propolis-mediated protection against oral pathogens, was assessed using ultra performance liquid chromatography- photo diode array detector- high resolution mass spectrometry (UPLC-PDA-HRMS) using reported method^54^.

**Figure 1:**
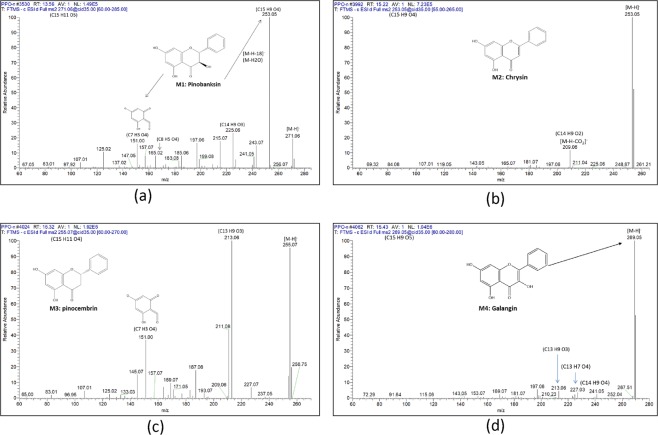
Fragmentation pattern of Pinobanksin (**a**), Chrysin (**b**), Pinocembrin (**c**) and Galangin (**d**).

Peak (M1) (Fig. 1a) [*m/z* 271.0606 (C_15_H_11_O_5_)^−^], UV_max_ 292, which was identified as pinobanksin, showed fragments at *m/z* 253 (C_15_H_9_O_4_)^−^ due to the loss of H_2_O, and *m/z* 151 (C_7_H_3_O_4_)^−^ due to ring C cleavage of flavonol. Peak (M2) (Fig. 1b) [*m/z* 253.0502 (C_15_H_9_O_4_)^−^], UV_max_ 269, 312, was assigned to chrysin, with an abundant peak at *m/z* 209 (C_14_H_9_O_2_)^−^ due to the loss of CO_2_. For peak (M3) (Fig. 1c) [*m/z* 255.0658 (C_15_H_11_O_4_)^−^], UV_max_ 290, the fragments at *m/z* 213 (C_13_H_9_O_3_)^−^ and *m/z* 151 (C_7_H_3_O_4_)^−^ were part of the pinocembrin flavanone fragmentation pattern. Peak (M4) (Fig. 1d) [*m/z* 269.0452 (C_15_H_9_O_5_)^−^], UV_max_ 267, 330, showed MS^2^ fragmentation at *m/z* 227 (C_13_H_7_O_4_)^−^ and 213 (C_13_H_9_O_3_)^−^, which were characteristic fragments of galangin.

### Characterization of niosomal vesicles

#### EE%

The data revealed that the EE% of PPE entrapped in niosomes was 91 ± 0.48%.

### Particle size, distribution and zeta potential of niosomal PPE

**Figure 2:**
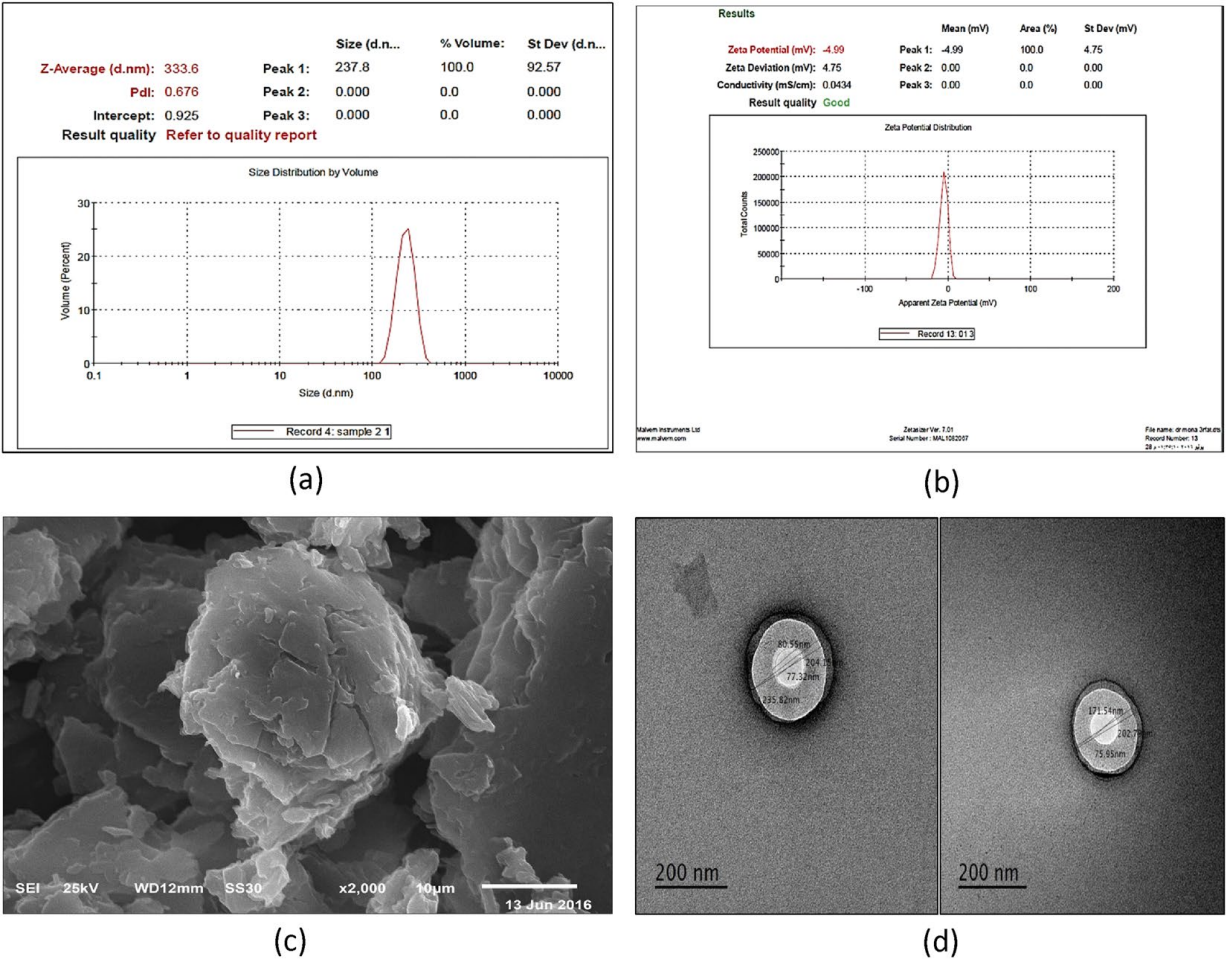
Particle size and PDI (**a**) Zeta potential (**b**) Scanning electron microscope (**c**) and Transmission electron microscopy (**d**) of niosomal propolis extract.

The results presented in Fig. 2a revealed an average particle size of 237–333 nm with a dispersant refractive index (RI) of 1.330 and a PDI of 0.676, with insignificant flocculation and viscosity (cP) equal to 0.8872. The results also showed a zeta potential of –4.99 with a negative charge on the surface (Fig. 2b).

#### SEM

SEM revealed the surface characteristics of niosomal PPE (Fig. 2c), which appeared to be smooth.

#### TEM

The morphology of the selected formula of PPE-loaded niosomes was investigated. The vesicle diameters of 172–235 nm were similar to the results obtained from Zetasizer. The results also revealed the presence of easily identified spherical multi-lamellar niosomes in a dispersed pattern (Fig. 2d).

### Physical features of the oromuco-adhesive film

The prepared oromuco-adhesive film containing PPE was regular and even in thickness and mass, with a uniform drug content. The film showed no visible fissures or wrinkles. The thickness of the formed film ranged from 0.29 ± 0.06 to 0.32 ± 0.17 mm, and the mass varied between 113.4 ± 4.3 and 115.5 ± 3.3 mg. The surface pH of the film was 6.8 ± 0.12. The film showed a drug loading efficacy of 90.48 ± 0.01%. All films had a satisfactory folding endurance of >300 and a muco-adhesive force of 0.42 ± 0.31 dyne/cm^2^. The swelling indices of the oromuco-adhesive PPE film as a function of time, with changes in diameter and weight, are shown in Table 2. There was a steady increase in the diameter (4-fold) and weight (3-fold) of the film during a period of 5 h.

**Table 2:** The swelling indices of the formed films.

Swelling index ± SD					
Time (h)	1	2	3	4	5
Diameter	20.01 ± 0.09	40.05 ± 0.17	55.36 ± 0.32	70.22 ± 0.08	80.73 ± 0.11
Weight	97.01 ± 0.19	129.49 ± 0.22	200.53 ± 0.05	268.58 ± 0.36	319.06 ± 0.28

### *In vitro* release of niosomal PPE from dispersions and films

Drug release from the formed films was governed by the volume growth of the polymeric matrix in the presence of phosphate buffer (pH 6.8), in addition to release through niosomes.

**Figure 3:**
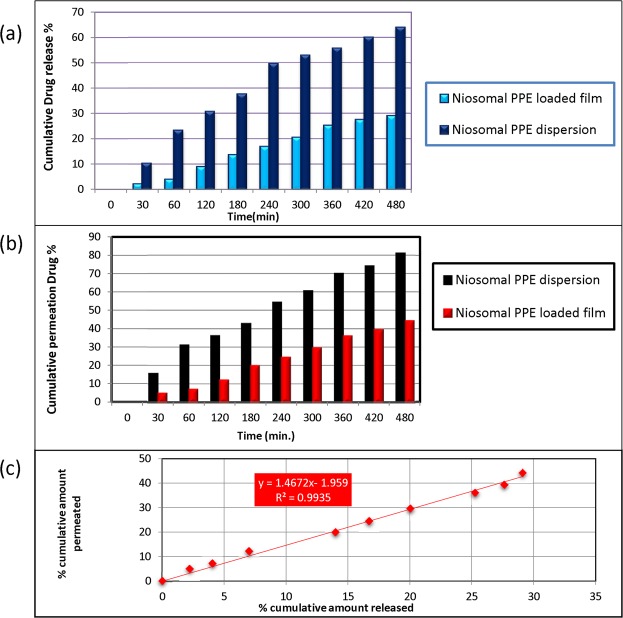
Release of PPE from niosomal PPE loaded film and niosomal PPE dispersion in phosphate buffer (pH 6.8) at 37 °C (**a**), Permeation studies through excised chicken pouch by niosomal PPE loaded film and niosomal PPE dispersion (**b**) and Correlation between the *in vitro* drug release and *ex vivo* drug permeation (**c**).

As shown in Fig. 3a, the drug release values from niosomal dispersions and oromuco-adhesive films were 64.05 ± 0.77% and 29.09 ± 0.13%, respectively, after 480 min. The drug release profiles of both formulations showed controlled drug delivery systems, with approximately 35% higher release in the niosomal dispersion than in the niosomal film.

### *Ex vivo* permeation

The *ex vivo* permeation values of PPE from the oromuco-adhesive film and the niosomal dispersion were 19.88 ± 0.09% and 42.88 ± 0.12%, respectively, after 180 min (Fig. 3b). The correlation coefficient (R) between drug permeation and drug release was 0.9967 (Fig. 3c). The permeation of the niosomal PPE oromuco-adhesive film indicated a flux of 155 µg/cm^2^/h, while the permeation of the niosomal PPE dispersion indicated a flux of 234 µg/cm^2^/h. The apparent permeability values of the niosomal PPE-loaded film and the niosomal PPE dispersion were 1.29 cms^−1^ and 1.95 cms^−1^, respectively.

### Kinetic release study

**Figure 4:**
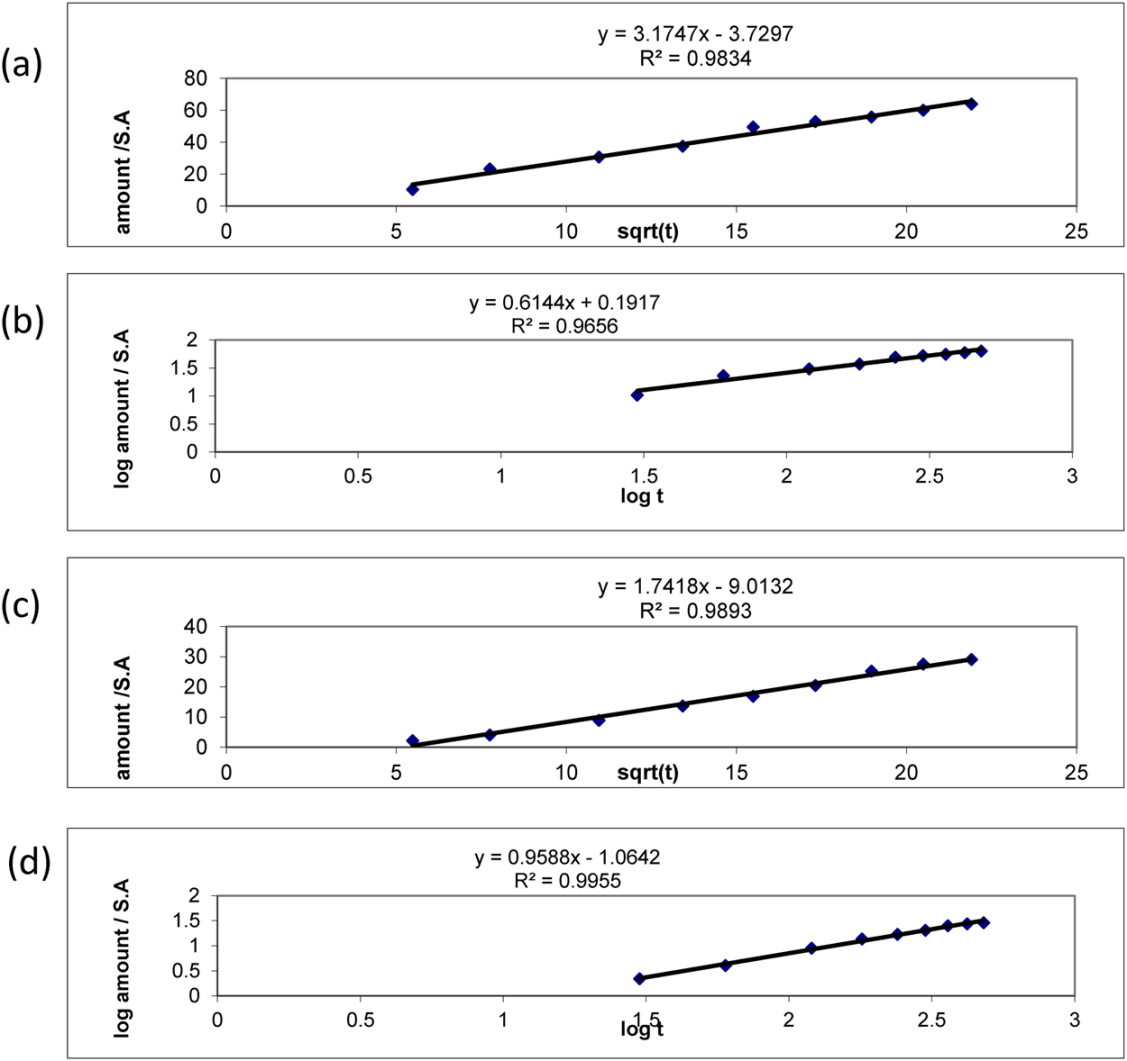
The kinetic release profiles of Higuchi model of niosomal PPE dispersion (**a**), Non-fickian permeation of niosomal PPE dispersion (**b**), Higuchi diffusion model of niosomal PPE loaded film (**c**) and Non-fickian permeation of niosomal PPE loaded film (**d**).

The Korsmeyer–Peppas equation and Higuchi kinetics were also used to analyse the release data for the tested film. The release rates ‘k’ and ‘n’ of the two formulae, F1 and F2, were computed by linear regression analysis using Microsoft Excel 2003 software. Correlation of determination (R^2^) values for the Higuchi and Peppas approaches were also calculated, compared and employed to estimate the precision of the fit^55–57^ (Table 3) (Fig. 4a,c).

**Table 3:** The kinetic release profiles of F1 and F2 formulae of PPE.

Formula	Higuchi diffusion		Korsmeyer-Peppas			Mechanism
	R^2^	Y	R^2^	Y	n	
F1	0.983	3.1747 × -3.7297	0.965	0.6144 × -0.1917	0.614	Non Fickian
F2	0.989	1.7418 × -9.0132	0.995	0.9588 × -1.0642	0.958	Non Fickian

In addition, the niosomal PPE dispersion and the niosomal PPE oromuco-adhesive film showed non-Fickian drug release (Table 3) (Fig. 4b,d).

### Clinical study of the niosomal PPE oromuco-adhesive film

A total of 24 patients suffering from RAUs participated in the present study. Twelve patients received the niosomal PPE oromuco-adhesive film (F2) (intervention group), and the second group received the film without the active component (placebo group). The trial ended when the outcomes were achieved. The intervention group included 4 males with a mean age of 23.8 ± 1.71 years and 8 females with a mean age of 22.9 ± 3.9 years. The placebo group included 4 males with a mean age of 22.8 ± 3.30 years and 8 females with a mean age of 24.6 ± 4.66 years, with no significant differences between the two groups. After application of the two formulations, the following parameters were assessed.

### Duration of adherence of the niosomal PPE oromuco-adhesive film

**Figure 5:**
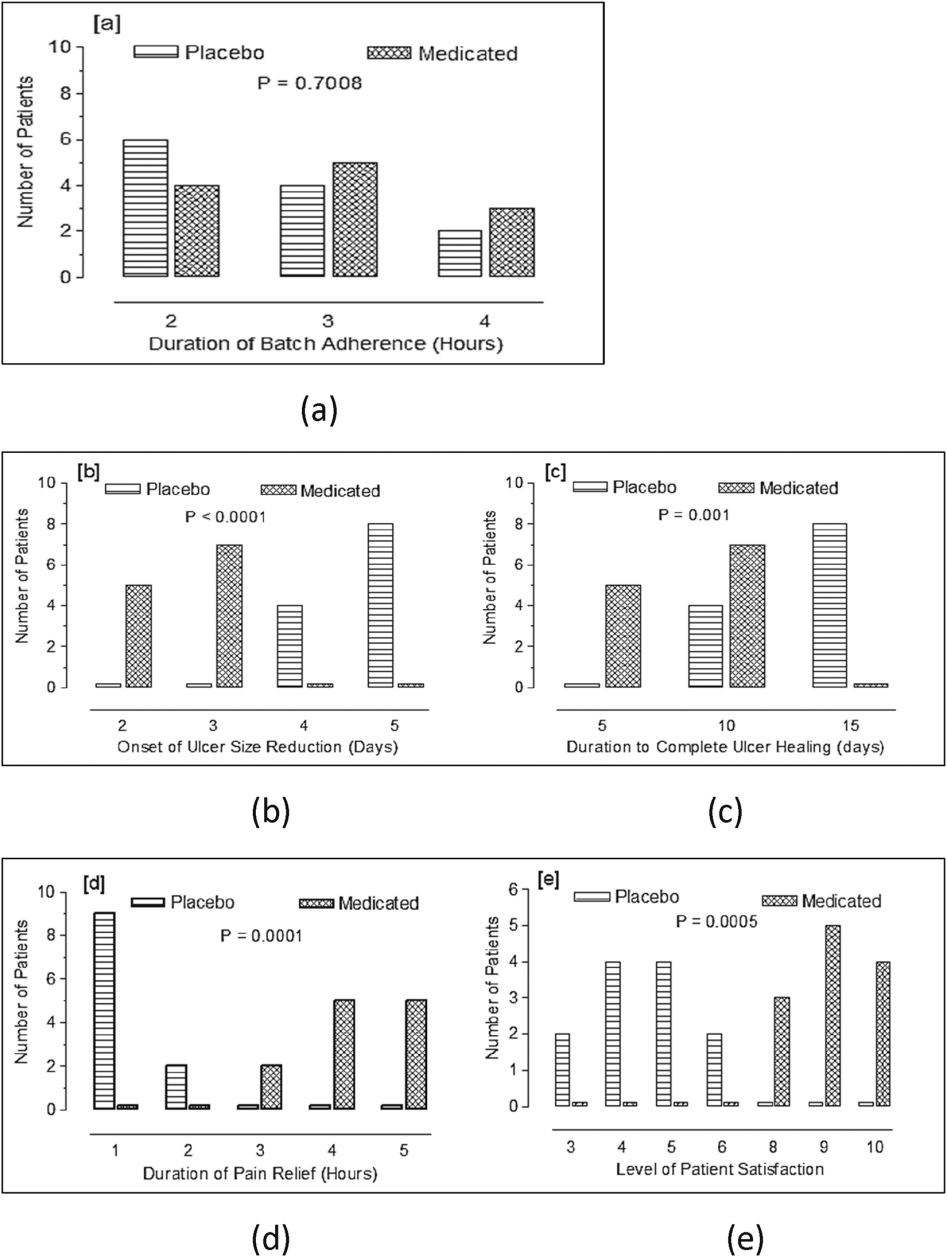
Application of non-medicated (Placebo) and propolis medicated auromuco-adhesive films twice daily in patients with aphthous ulcers. The number of patients used was 12 in each group. Results were compared using Chi square test.The study includes Duration of adherence (**a**), Onset of ulcer size reduction (**b**), Duration of complete ulcer healing (**c**), Duration of pain relief (**d**) and the Level of patient satisfaction (**e**). P < 0.05 was considered significant.

No significant difference was found between the intervention group and the placebo group (p = 0.7008). The duration of film adherence was 2 h in 4 patients, 3 h in 5 patients and 4 h in 3 patients in the intervention group, while this value was 2 h in 6 patients, 3 h in 4 patients and 4 h in 2 patients in the placebo group (Fig. 5a).

### Onset of ulcer size reduction

When the onset of ulcer size reduction was evaluated, a statistically significant difference was found between the intervention and placebo groups (p < 0.0001). In the intervention group, there was a significant reduction in ulcer size on the second day of treatment in 5 patients and on the third day of treatment in 7 patients, while for the placebo group, no decrease in ulcer size was observed on these days, and a size reduction was observed only on the fourth day of treatment in 4 patients and on the fifth day of treatment in 8 patients (Fig. 5b).

### Time to complete ulcer healing

**Figure 6:**
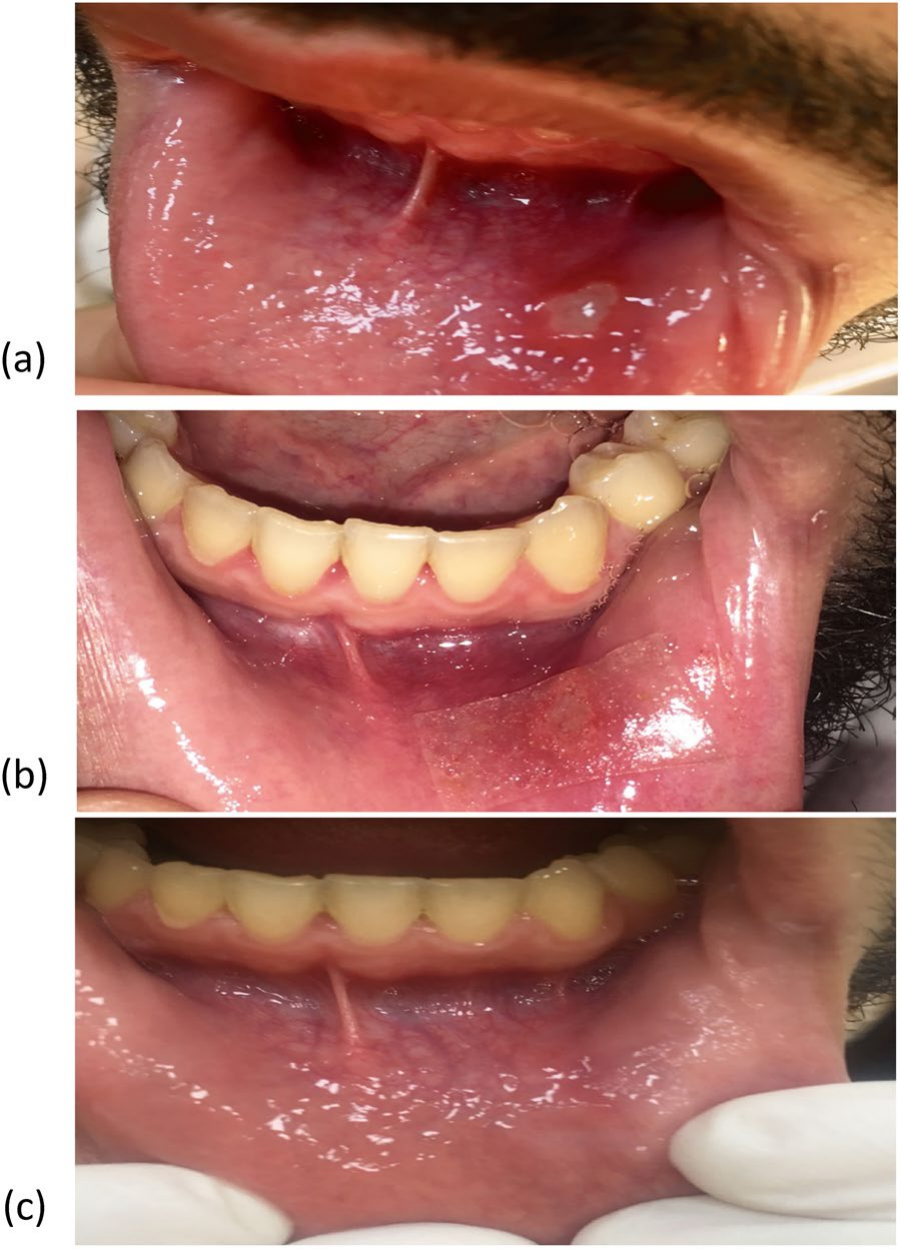
Photograph of one patient with aphthous ulcer treated with the propolis-medicated formula twice daily before treatment (**a**), 2 days after treatment (**b**) and healing of the ulcer 4 days after treatment (**c**).

The intervention group showed a significantly shorter time to complete ulcer healing than the placebo group (p = 0.001). Five patients from the intervention group recorded complete ulcer healing during the first 5 days, and 7 patients recorded complete ulcer healing within the period from day 5 to day 10 of treatment (Figs 5c and 6). On the other hand, no patients in the placebo group recorded healing of ulcers during the first 5 days of treatment, while 4 patients recorded complete ulcer healing within the period from day 5 to day 10 of treatment, and 8 patients recorded complete ulcer healing from day 10 to day 15 of treatment.

### Duration of pain relief

A highly significant difference in the duration of pain relief was found between the intervention group and the placebo group (p = 0.0001). In the intervention group, pain was relieved after almost 3 h in 2 patients, after 4 h in 4 patients and after 5 h in 4 patients. In contrast, in 9 patients in the placebo group, pain relief lasted for only one hour, and pain relief lasted for 2 h in only 2 patients (Fig. 5d).

### Level of patient satisfaction

The intervention group was significantly more satisfied than the placebo group (p = 0.0005). All patients in the intervention group recorded high scores of 8 to 10, with 3 patients recording a score of 8, 5 patients recording a score of 9, and 4 patients recording a score of 10. In the placebo group, 2 patients recorded a score of 3, 4 patients recorded a score of 4, 4 patients recorded a score of 5, and only 2 patients recorded a score of 6 (Fig. 5e).

## Discussion

Flavonoids, phenolic acids and their esters, terpenes and fatty acids are considered to be the major classes in all propolis samples. The detailed chemical profile, however, varies according to the plants surrounding the beehive. Propolis preparations obtained from temperate regions are considered to have similar compositions, with the main compounds being flavonoids, such as pinocembrin, pinobanksin, chrysin and galangin^58^.

The fragmentation of propolis in the present study matched reports from a previous work^59^. Pinocembrin and galangin, followed by chrysin, were the major flavonoids identified in this propolis sample, which could contribute to its observed potent antibacterial activity.

The amount of entrapped PPE in the formed niosomes was governed by the cholesterol content, which prevented the conversion of the gel of the niosomal system into liquid; thus, the encapsulation of hydrophilic drugs was enhanced. Cholesterol prevented leaking in the bi-layer membranes, making the membrane firmer, as the surfactants were more condensed during filling^60^. In addition, the EE% of PPE was affected by the method of free drug separation; in this study, that process was carried out by refrigerated centrifugation, resulting in a substantial increase in PPE trapped inside the niosomes. Additionally, this mechanism explained the events that occurred during the freeze-thawing cycle. The drug and vesicles were concentrated during freezing, and the particles were closely packed with each other, resulting in the merging of niosomal vesicles that efficiently entrapped PPE^61^. Finally, niosomes prepared from Span 60 were superior to those prepared from other surfactants. Span 60 has the highest phase transition temperature^62^. The longer saturated alkyl chain of Span 60 produces high EE% of the drug and consequent stability of the niosomes^63,64^. Finally, the reverse-phase evaporation method has unique advantages for encapsulating water-soluble materials, such as PPE. With the reverse-phase evaporation method, the organic solvent is simply removed from the inverted micelles, resulting in vesicles with larger aqueous space-to-lipid ratios and consequently higher EE% values^65^.

The obtained sizes of the formed niosomes were in the optimal nano-range and were consistent with the results obtained by TEM, while the polydispersity index (PDI) indicated that the particles were homogenous, with an optimum viscosity for topical application.

The zeta potential values revealed the presence of a stabilization mechanism to counteract the electrostatic repulsion of the formed niosomes, while SEM revealed a morphology that was defined by the filling effect of the surfactant, in addition to deposition in thicker layers at deeper invaginations. After dissolution of all components in the solvent and evaporation of the solvent, the components crystallized on top of the new surface, which may have caused some of the fine crystalline structures to disappear, creating niosomes with a smooth surface.

After incorporating niosomes into the oromuco-adhesive film, the swelling indices of the prepared film could be due to the presence of hydrophilic polymers that form a network assembly through hydrogen bonds with water molecules^66^. The greater swelling of HPMC may be due to the presence of sponge-like pores in the inner layers of HPMC, which allows rapid entry of the PBS solution into the polymer network^67^ with the presence of the water insoluble polymer Eudragit. In addition, the presence of PG would alter the water distribution within such systems by modifying the matrix structure. The film did not show any noticeable alterations in shape and retained its integrity during the investigation period. It is important to mention that the films have considerable muco-adhesive force as they contain HPMC and PVA^68,69^. Thus, it can be deduced that in addition to its role as a film-forming polymer, PVA also acts as a synergistic agent that augments the muco-adhesiveness of HPMC. Moreover, Eudragit polymers affect the bioadhesive force of PVA.

The *in vitro* release results suggested that the presence of niosomes in the films mediates the delivery and controlled release of the efficiently trapped propolis over a sustained period of time. The hydration and swelling behaviour of the formed film can affect the drug release profile because diffusion, swelling and erosion are the mechanisms that control drug release^70^. Controlled drug release can be interpreted as being governed by erosion/diffusion mechanisms. The rate of drug release could be explained by the higher swelling of HPMC, which had a direct effect on drug release. The stronger the swelling of the polymers is, the slower the release of the drug^71^.

*Ex vivo* results showed that PPE was released from the formulations, permeated through the membrane, and thus had the potential to permeate through human oromucosal tissues. Furthermore, the good correlation between *in vitro* drug release and *ex vivo* drug permeation suggests efficient transport of propolis released from the niosomal films across the chicken pouch tissue. This effect may be due to the adherence of the hydrated muco-adhesive films to the surface of the mucosal tissue for a sufficient period of time, with subsequent drug release from niosomes supporting drug availability and a high concentration diffusion gradient across the membrane^72^, in addition to the penetration-enhancing effect of the lipid structure of the niosomes and PG. The obtained results were also consistent with the results of many investigations that showed that direct and prolonged contact between vesicles and mucosal tissues supports effective drug delivery. This attribute may be due to the inclusion of nonionic surfactants in the structure. Surfactants affect the overall penetration enhancement by being adsorbed at boundaries, interacting with biological membranes and changing stratum corneum barrier function^73–75^.

Kinetic studies were conducted to investigate the drug release pattern explained by the Higuchi model. When the films swelled, solvent pathways were created, through which the drug diffused and was released from the eroded matrix. Consequently, the relative combination of erosion and diffusion will dominate the overall release mechanism. Further kinetic studies revealed that the drug release was consistent with non-Fickian diffusion.

The prepared films containing niosomal PPE were used to treat a group of patients suffering from aphthous ulcers. There was a significant decrease in ulcer size in the propolis-treated group, in which all patients recorded the onset of ulcer size reduction after only one to two days of treatment. On the other hand, the placebo group showed a delayed reduction of ulcer size starting on the fourth day of treatment. This finding reflects the positive effect of propolis on ulcer size reduction; these results are consistent with a previous study^37^, in which the clinical effectiveness of propolis-containing buccal pastes in different formulations with sesame oil and olive oil was examined, and the obtained result indicated that both formulations caused a significant decrease in ulcer size in propolis-treated subjects.

Improved healing time in propolis-treated patients was also reported^31^; the efficacy of daily use of 500 mg propolis capsules by patients with RAUs was evaluated for 3 months, and the results showed a significant decrease in healing time in propolis-treated patients. Figure 6 shows a picture of one patient treated with the medicated formula.

Pain relief lasted much longer in the intervention group than in the placebo group. This result is due to the anti‐inflammatory effect of propolis and was consistent with the results of many studies^37,76^.

The pain relief reported in the placebo group, though brief, may be attributed to the protection of the oral ulcer, as the film forms an insulating layer that protects the aphthous ulcer from all sources of irritation in the oral cavity, whether mechanical or chemical due to various food constituents.

Regarding patient satisfaction, in contrast to the dissatisfied placebo group, the patients in the intervention group were greatly satisfied with the effect of the propolis oromuco-adhesive film and with its effects on their quality of life, as the propolis film reduced the onset of ulcer size reduction, prolonged the duration of pain relief and accelerated the healing time of RAUs. The positive impact of propolis could be due to enhancement of the immune system of the patients.

The efficacy of propolis as a treatment for RAUs was previously reported for many dosage forms, such as topical creams, oral capsules, topical solutions, and buccal pastes^13,15,31,37,76,77^, but propolis has never been applied in the form of an oromuco-adhesive film, as reported in the present study. Although previous dosage forms presented good effects, they required frequent application, which might lead to less patient compliance. In the present study, the propolis oromuco-adhesive film was applied only twice daily, and it resulted in significant improvements in all aspects of the disease and produced substantial enhancement of patient quality of life.

## Conclusion

In the present study, a new generation of oromuco-adhesive films containing niosomal propolis was presented to employ the diverse biological properties of propolis in a controlled-release drug delivery system. The active ingredient was maintained in the oral cavity for a prolonged period of time. The films produced very satisfactory outcomes in patients with RAUs in terms of ulcer size reduction, prolonged duration of pain relief and reduced ulcer healing time, which ultimately resulted in a very high level of patient satisfaction, proving the success of this new drug delivery system.

## Data Availability

All data generated or analyzed during this study are included in this article.
