# Publisher Correction: Propolis-based niosomes as oromuco-adhesive films: A randomized clinical trial of a therapeutic drug delivery platform for the treatment of oral recurrent aphthous ulcers

**DOI:** 10.1038/s41598-020-59349-w

**Published:** 2020-02-07

**Authors:** Mona G. Arafa, Dalia Ghalwash, Dina M. El-Kersh, M. M. Elmazar

**Affiliations:** 1 Department of Pharmaceutics and Pharmaceutical Technology, Faculty of Pharmacy, The British University in Egypt (BUE), El-Sherouk city, Cairo 11837 Egypt; 2 Department of Oral Medicine and Periodontology, Faculty of dentistry, The British University in Egypt (BUE), El-Sherouk city, Cairo 11837 Egypt; 3 Department of Pharmacognosy, Faculty of Pharmacy, The British University in Egypt (BUE), El-Sherouk city, Cairo 11837 Egypt; 4 Department of Pharmacology, Faculty of Pharmacy, The British University in Egypt (BUE), El-Sherouk city, Cairo 11837 Egypt; 5 Chemotheraputic Unit, Mansoura University Hospitals, Mansoura, 35516 Egypt

Correction to: *Scientific Reports* 10.1038/s41598-018-37157-7, published online 21 December 2018

The original version of this Article contained an error in Affiliation 3, which was incorrectly given as ‘Department of Pharmacognosy, The British University in Egypt (BUE), El-Sherouk city, Cairo, 11837, Egypt’. The correct affiliation is listed below:

Department of Pharmacognosy, Faculty of Pharmacy, The British University in Egypt (BUE), El-Sherouk city, Cairo, 11837, Egypt

This error has now been corrected in the HTML and PDF versions of the Article.

